# In Silico Hybridization and Molecular Dynamics Simulations for the Identification of Candidate Human MicroRNAs for Inhibition of Virulent Proteins' Expression in *Staphylococcus aureus*

**DOI:** 10.1002/jcb.30684

**Published:** 2024-12-10

**Authors:** Harshita Tiwari, Subhadip Saha, Monidipa Ghosh

**Affiliations:** ^1^ Department of Biotechnology National Institute of Technology Durgapur India

**Keywords:** MD simulations, miRNA, virulent proteins

## Abstract

*Staphylococcus aureus* is a major threat to human health, causing infections that range in severity from moderate to fatal. The rising rates of antibiotic resistance highlight the critical need for new therapeutic techniques to combat this infection. It has been recently discovered that microRNAs (miRNAs) are essential for cross‐kingdom communication, especially when it comes to host‐pathogen interactions. It has been demonstrated that these short noncoding RNAs control gene expression in the gut microbiota, maintaining homeostasis; dysbiosis in this system has been linked to several diseases, including cancer. Our research attempts to use this understanding to target specific bacterial species and prevent severe diseases. In particular, we look for putative human miRNAs that can attach to virulent bacterial proteins' mRNA and prevent them from being expressed. In‐silico hybridization experiments were performed between 100 human miRNA sequences with varied expression levels in gram‐positive bacterial infections and five virulence factor genes. In addition, these miRNAs' binding properties were investigated using molecular dynamics (MD) simulations. Our findings demonstrate that human miRNAs can target and inhibit the expression of bacterial virulent genes, thereby opening up new paths for developing innovative miRNA‐based therapeutics. The implementation of MD simulations in our study not only improves the validity of our findings but also proposes a new method for constructing miRNA‐based therapies against life‐threatening bacterial infections.

## 1 Introduction

*Staphylococcus aureus* is a prevalent bacterium that can coexist harmlessly with the host and may appear benign at first, but it can become opportunistic and cause a variety of serious infections including meningitis, endocarditis, septicemia, pneumonia, osteomyelitis, and so forth [1, 2]. Its ability to produce toxins, form biofilms, and evade host immune responses, frequently necessitates lengthy antibiotic treatments, which are made more difficult by the generalized resistance to antibiotics [3]. Furthermore, sepsis is caused by a multitude of inflammatory responses that are triggered both locally and systemically by staphylococcal pyogenic infection [4]. *S. aureus*‐related sepsis still has a 30% fatality rate, with rates as high as 90% in multiorgan and shock failure patients, despite advances in treatment [5]. It has come to light in recent years how uniquely miRNA is regulated in a variety of biological activities. The class of short, noncoding RNAs known as miRNAs is extensively present in eukaryotes and ranges in length from 19 to 25 nucleotides. MiRNAs are important components of intracellular signaling and can negatively control target genes by inhibiting mRNA transcription and translation [6, 7]. Prior studies have shown that miRNAs are involved in controlling bacterial infections as well as being used by bacteria for self‐survival [8, 9, 10, 11, 12]. Although earlier research has indicated that miRNAs may be viable candidates for Host Directed Therapy (HDT) in bacterial infections, the field of miRNAs for HDT is still in its infancy [11, 12]. Several studies indicate the regulation of miRNAs in the host body during *S. aureus* infection. Research examining the effect of *Staphylococcal* enterotoxin B (SEB) on microRNA (miRNA) expression has demonstrated that SEB significantly upregulates the expression of miR‐155, which in turn contributes to aberrant inflammatory reactions. *S. aureus*‐infected macrophages have been shown to have decreased miR‐24 expression, which supports a pro‐inflammatory phenotype [13, 14]. Furthermore, the colonization of *S. aureus* in long‐term skin wounds increases the expression of miR‐15b‐5p, which hinders the healing process [15, 16]. A complex interaction between miRNAs and the dynamics of *S. aureus* infection is suggested by the fact that deletion of miR‐223 improves wound repair and impaired wound healing and altered cytoskeletal function are linked to mice lacking miR‐142 [17, 18].

A fresh perspective for treating severe bacterial infections has been provided by several fascinating research that show changes in bacterial genes in parallel with host gene alterations during infection. Extracellular vesicles (EVs) carrying miRNAs that control gene expression are involved in intercellular communication [19, 20]. These EVs, which can be found in a variety of biological fluids, bind with the target cells to change the expression and function of genes [21]. According to recent investigations, eukaryotic miRNAs alter the proliferation and abundance of bacteria, suggesting that cross‐kingdom RNA interference via EVs plays a role in host–pathogen communication [22, 23, 24]. Prior studies have provided sufficient evidence of the transfer of miRNAs from intestinal epithelial cells to the gut microbiota, whereby they function as growth regulators for the microbiome [25, 26]. The transfer method of miRNA let‐7b‐5p to *Pseudomonas aeruginosa*, the etiological agent of respiratory infections, via EVs, has been clarified by a recent work. Important genes including *Clv1*, *Ppk1*, and *AmpC* are downregulated as a result of this process, which increases susceptibility to antibiotics and reduces the production of biofilms [27]. Chakrabarti et al., have discovered putative human miRNAs that can bind to *P. aeruginosa* mRNA and prevent its production. They used in‐silico hybridization procedures to evaluate differentially expressed human miRNAs in response to *P. aeruginosa* infection against common pathogenicity factors [28].

*S. aureus* uses a variety of surface proteins and regulators to increase its pathogenicity. The *spa* gene encodes protein A, which binds to immunoglobulin such as IgG and disrupts opsonization and phagocytosis, so enabling *S. aureus* to elude the host immune response [29, 30]. The binding of Clumping Factor B (ClfB) to Cytokeratin 10 is essential for nasal colonization and the development of cutaneous abscesses [31, 32]. HtrA1 and HtrA2, two HtrA‐like serine proteases, are involved in the stress response and virulence, respectively. HtrA2 facilitates the breakdown of host proteins [33, 34]. MgrA acts as a global regulator, regulating the development of biofilms, resistance to antibiotics, and immunological evasion [35, 36, 37]. SarA, however, affects several virulence factors, such as the inhibition of toxic shock syndrome toxin‐1 (TSST‐1). Each of these components helps *S. aureus* resist human defenses and cause a variety of illnesses [38]. In this study, we aim to find out candidate human miRNAs that may bind with these virulent protein's mRNA and cause inhibition of their expression. To find human miRNAs that are involved in gram‐positive bacterial infections, particularly, those caused by *S. aureus*, we first carried out a thorough literature search. Next, we used in‐silico hybridization analysis to test these miRNAs against *S. aureus's* known pathogenic proteins. In addition, these miRNAs' binding properties were investigated using molecular dynamics (MD) simulations. Surprisingly, miRNAs with low hybridization energy and strong binding to *S. aureus* mRNA sequences have surfaced as viable treatments for treating severe *S. aureus* infections. To treat this disease, the goal of this work is to find interactions between miRNAs and *S. aureus* mRNAs. To tackle *S. aureus* infections, this combination approach offers a feasible route for the development of innovative miRNA‐based therapeutics.

## 2 Methods and Materials

### 2.1 Identification and Extraction of miRNA and mRNA Sequences

The 3′ and 5′ untranslated region (UTR) sequences of five pathogenic protein‐encoding mRNAs including Protein A, ClfB, HtrA, SarA, and MgrA from *S. aureus* were extracted after meticulous evaluations from the vast resources of the National Center for Biotechnology Information (NCBI) GenBank (http://www.ncbi.nlm.nih.gov) [39]. Concurrently, sequences of 100 human miRNAs that are involved in gram‐positive bacterial infection were retrieved from reputable sources including TargetScan (v7.0) (https://www.targetscan.org/vert_80/), miRBase v.22 ( http://www.mirbase.org/) [40, 41]. The foundation for later investigations, which sought to clarify the regulatory dynamics underpinning microbial pathogenicity and gene expression regulation, was established by this thorough search and retrieval procedure.

### 2.2 Target Prediction for Virulent Factors

Advanced computational algorithms were used to anticipate miRNA binding sites within the transcripts of five target genes to uncover potential regulatory interactions between miRNAs and mRNAs. With the aid of two well‐known platforms that are highly regarded for their precision in RNA–RNA interaction prediction RNA hybrid (http://bibiserv.techfak.uni-bielefeld.de/rnahybrid .) and IntaRNA (http://rna.informatik.uni-freiburg.de/IntaRNA) [42, 43]. Both RNAhybrid and IntaRNA are useful tools for predicting RNA–RNA interactions; however, IntaRNA prioritizes quick and precise RNA–RNA hybrid prediction with improved parameterization and adjustable control over prediction modes and output formats, while RNAhybrid concentrates on predicting multiple potential binding sites of miRNAs in large target RNAs with specific features like preventing G: U base pairs. The complementary base pairing between miRNAs and their putative binding sites on target mRNAs was examined with help of these high‐throughput techniques [43]. We were able to pinpoint putative regulatory motifs and estimate the probability of miRNA‐mediated posttranscriptional gene regulation, providing insight into the complex regulatory networks controlling the dynamics of gene expression in our study system.

### 2.3 Structure Prediction of Interacting RNA Molecules (miRNA–mRNA Complex)

Most of the miRNA target prediction methods rely mainly on predicting the secondary structure of RNA duplexes. The secondary structure was predicted using the RNAfold web server (http://rna.tbi.univie.ac.at/) to verify the folding affinity between the selected miRNAs and their corresponding target genes. If the synthesis of the miRNA–mRNA duplexes is energetically advantageous, it suggests that the gene targets are functioning [44, 45]. The miRNA–mRNA duplex result was supplied in a dot‐bracketed style, and RNAComposer (http://rnacomposer.ibch.poznan.pl and http://rnacomposer.cs.put.poznan.pl/) was utilized to estimate the tertiary structure [46].

### 2.4 MD Simulation

The miRNA–mRNA hybrids obtained were applied to robust MD simulation to further ascertain the stability and structural alterations of the complexes. Previously, MD simulations have been applied for fixing steric clashes in computationally developed RNA models, evaluating the dynamics, as well as to investigate the interaction between RNA and other molecules making it an essential tool in RNA biology [47]. Previously MD was used to analyze the interaction between the *Caenorhabditis elegans* let‐7 miRNA fragment and complementary site *LCS*2 located in the 3' UTR of mRNA lin‐41 [47].

GROMACS version 2023.1 was applied to study mRNA–miRNA dynamics. GROMACS is a freely available and open‐source tool for molecular simulations of various biomolecules [48]. Briefly, the topology of miRNA–mRNA duplexes was prepared by AMBER94 force field using the pdb2gmx function of GROMACS. The duplex was solvated with TIP3P water model and placed in a cubic box. The entire system was neutralized by desired number of Na^+^ and Cl^−^ counter ions. The output file was energy minimized by steepest descent with a maximum number of steps limited to 50 000. The energy‐minimized systems were applied to equilibration utilizing NPT followed by NVT ensembles. The V‐rescale thermostat was used with coupling time of 0.1 ps for maintaining constant temperature of 300 K during 200 ps NVT run [49]. For a 400 ps NPT, Parrinello–Rahman pressure coupler was applied to maintain pressure at 1.0 bar. Periodic boundary conditions were established and for long‐range interaction electrostatic Particle Mesh Ewald were set [50]. All bond lengths including hydrogen atoms were constrained by the LINCS algorithm. A 50 ns of final production MD was run in NPT mode with the time step set to 2 fs.

## 3 Results

### 3.1 In‐Silico Hybridization of Human miRNAs With *S. aureus* mRNA

Bioinformatics approaches are critical tools for narrowing down and identifying candidate miRNA targets while also supplementing wet‐lab biological validation. However, predicting miRNA targets is difficult due to miRNAs' small size and poor complementarity to the target, which prevents the use of standard sequence alignment procedures [51, 52]. MiRNA target prediction algorithms use a variety of features to select viable targets and avoid false positives. The presence of a seed match and the free energy of the RNA–RNA hybrid are the two most widely employed properties. The seed match follows established miRNA target recognition guidelines, with Nucleotides 2–8 of the miRNA 5′ end critical for its function [53]. The prediction score is influenced by the number of Watson–Crick pairs (6mer, 7mer, or 8mer) that exist between the miRNA seed sequence and the target. Noncanonical miRNA binding and compensatory pairing at the 3′ end of the miRNA can also help to improve the ranking of projected targets [54, 55]. Some target prediction techniques include the stability of the miRNA–mRNA hybrid by computing theoretical Gibbs free energy values and including them in the ranking score of potential targets [56].

**Figure 1:**
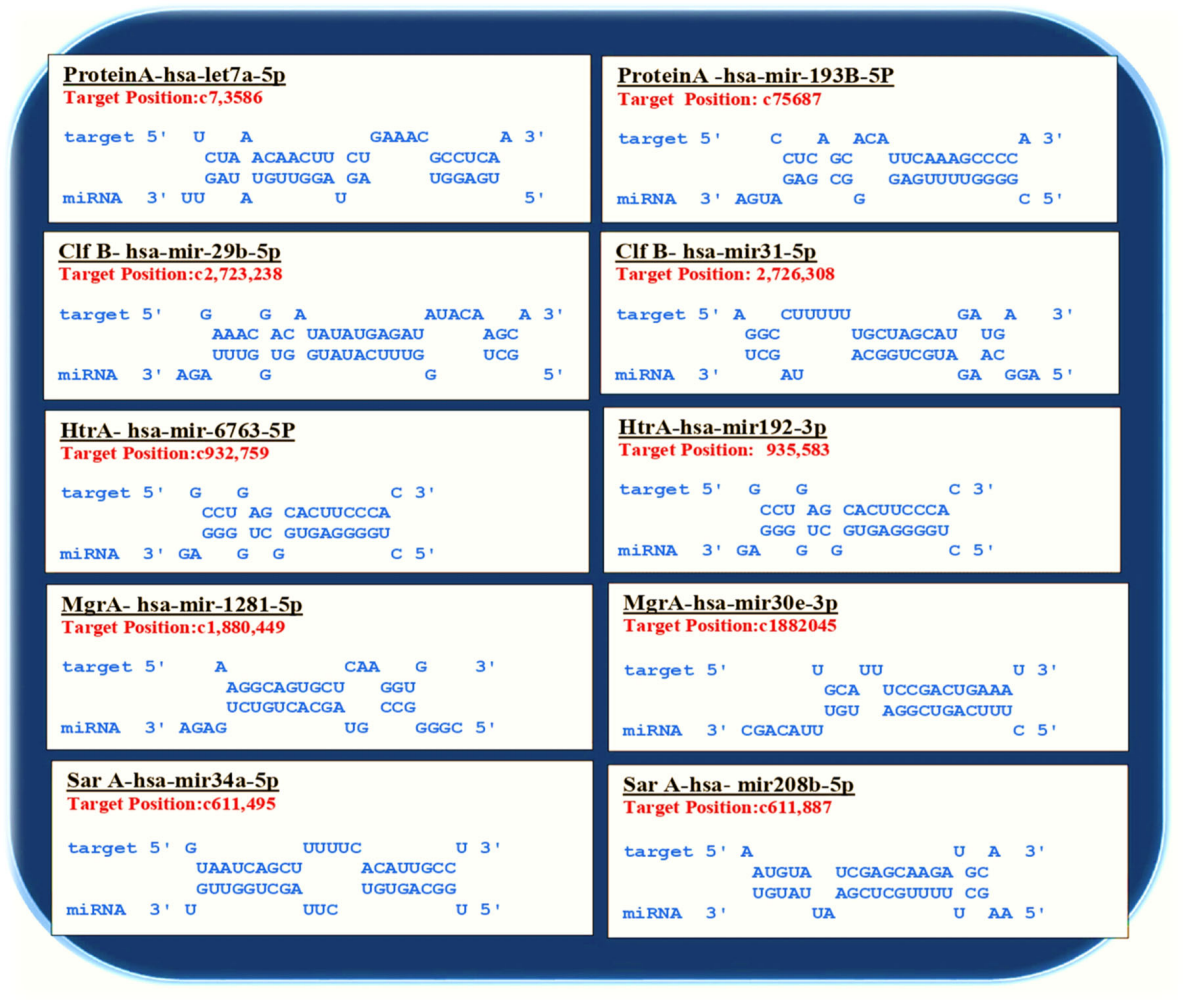
Seed pairings sequence, the target sites, and hybridization patterns of miRNAs with mRNAs of *Staphylococcus aureus* which determines stability of the seed–target duplex.

In‐silico hybridization was performed between human miRNAs with varied expression levels in gram‐positive bacterial infections and *S. aureus* virulence factor genes. In this study, 500 bp sequences from the 3′ UTR and 5′ UTR regions of five *S. aureus* virulence factor genes were employed in in‐silico hybridization experiments with 100 human miRNA sequences. This strategy sought to identify miRNAs that could suppress bacterial virulence factors. Several positive hybridization cases were discovered, and several miRNAs had low hybridization energy, indicating a strong binding affinity. To discover significant miRNA–mRNA interactions, two energy thresholds were set: RNAhybrid (−22 kcal/mol) and IntaRNA (−9 kcal/mol). Figure 1 illustrates the target positions of human miRNA in bacterial gene and seed pairings miRNA–mRNA duplex, and shows that the majority of miRNA–mRNA combinations have seed sequences longer than six nucleotides, suggesting that they have a high chance of being effective targets for *S. aureus*. This is supported by the energies shown in Table 1, which presents the energies calculated by RNAhybrid and IntaRNA and shows that miRNAs are energetically advantageous options for addressing *S. aureus*. hsa‐mir‐193b‐5p showed strong hybridization with the ProteinA mRNA, with a combined free energy E of −18.46 kcal/mol, a hybridization energy of −21.49 kcal/mol for IntaRNA, and minimum free energy (mfe) of −29 kcal/mol for RNAhybrid followed by hsa‐mir‐128‐1‐5p and hsa‐mir‐30e‐3p which showed strong hybridization with MgrA mRNA both showed good binding energies with a combined free energy E of −10 and −15 kcal/mol, a hybridization energy of −18.6 and 16.58 kcal/mol for IntaRNA, and a mfe of −26.3 kcal/mol and −23.9 for RNAhybrid. Other putative miRNAs including hsa‐mir‐34a‐5p, hsa‐mir‐192‐3p, and so forth also had low binding energies that fell below the predefined criterion, implying that they may decrease gene expression and, hence, reduce *S. aureus'* ability to survive and spread disease. On the other hand, the two miRNAs hybridizing with ClfB mRNA, hsa‐mir‐29‐1‐5p and hsa‐mir‐31‐5p, showed the least efficacy, with mfe for RNA hybridization at −23.7 and −22.3 kcal/mol, respectively for RNAybrid. IntaRNA's hybridization energies were −12.01 and −15.52 kcal/mol, whereas the overall energies were −9.2 and −9.99 kcal/mol for miRNAs hybridizing with ClfB mRNA. According to these findings, these miRNAs are not as good in targeting the gene. According to our research, more research should be done on the potential benefits of miRNA‐based therapy for lowering Gram‐positive bacterial infections.

**Table 1:** The table displays the mRNA–miRNA pairs along with their binding energies.

SI. no	Gene–miRNA	Mfe (kcal/mol) RNAhybrid)	*E* (kcal/mol) (IntaRna)	Hybridization energy (kcal/mol) (IntaRna)
	PTEN 3′utr**:**hsa‐mir‐21‐5p (control)	−23.4	−10.69	−13.74
1.	Protein A 3′ UTR: hsa‐let‐7a‐5p	−22.4	−12.28	−13.58
2.	Protein A 5′ UTR: hsa‐mir‐193b‐5p	−29	−18.6	−21.49
3.	ClfB‐3′ UTR**:** hsa‐mir‐29‐1‐5p	−23.7	−9.2	−12.01
4.	ClfB‐5′ UTR**:** hsa‐mir‐31‐5p	−22.3	−9.99	−15.52
5.	HtrA5′ UTR**:** hsa‐mir‐6763‐5p	−27.3	−11.35	−19.09
6.	HtrA3′ UTR**:** hsa‐mir‐192‐3p	−28.4	−11.68	−16.58
7.	MgrA3′ UTR**:** hsa‐mir‐128‐1‐5P	−26.3	−10.52	−18.6
8.	MgrA5′ UTR**:** hsa‐mir‐30e‐3P	−23.9	−15	−17.37
9.	SarA 5′ UTR**:** hsa‐mir‐34a‐5P	−28	−11.62	−21.54
10.	SarA 3′ UTR**:** hsa‐mir‐208b‐5P	−23.2	−13.07	−17.18

### 3.2 miRNA–mRNA Duplex's Secondary Structures and Tertiary Structures Predictions

**Figure 2:**
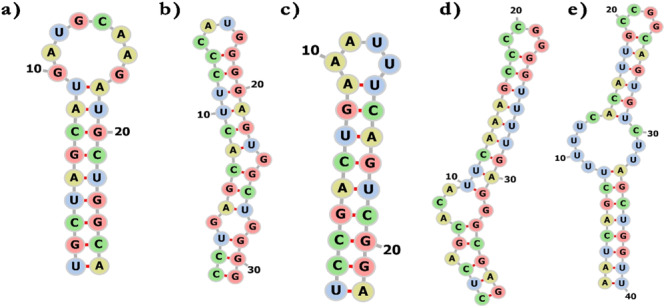
Secondary structures of miRNA–mRNA duplex. (a) ClfB‐5' UTR**:** hsa‐mir‐31‐5p, (b) HtrA‐5′ UTR: hsa‐mir‐6763‐5p, (c) MgrA‐5'UTR**:** hsa‐mir‐30e‐3p, (d) Protein A‐5′ UTR: hsa‐mir‐193b‐5p, and (e) SarA‐5'UTR: hsa‐mir‐34a‐5p.

**Figure 3:**
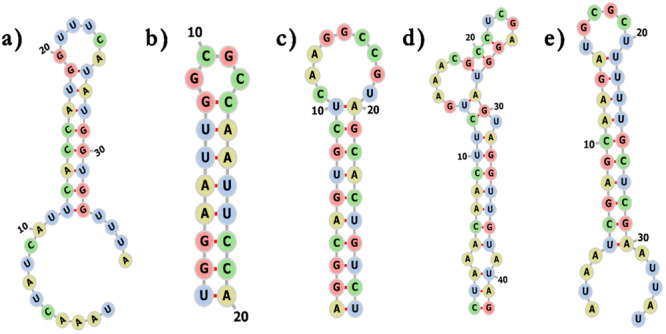
Secondary structures of miRNA–mRNA duplex. (a) ClfB‐3′ UTR: hsa‐mir‐29‐1‐5p, (b) HtrA‐3′ UTR: hsa‐mir‐192‐3p, (c) MgrA‐3′ UTR: hsa‐mir‐128‐1‐5p, (d) ProteinA‐3′ UTR: hsa‐let‐7a‐5p, and (e) SarA‐3′ UTR: hsa‐mir‐208b‐5p.

The secondary structure of human miRNA was examined by using enlarged thermodynamic parameters for sequence dependency and the RNAfold web service to determine the secondary structure of miRNA–mRNA duplex [57]. To prevent isolated base pairs—which are essentially single base‐pair helices that have the potential to disrupt the structure—the folding method that was chosen took into account both the partition function and the mfe. By using this method, the RNAfold program was able to determine which secondary structures were the most reliable and consistent. We examined the anticipated human miRNA folding patterns to comprehend the interactions between bacterial mRNA and human miRNA. Finding possible interactions and targeting mechanisms required careful consideration, which this investigation provided. The 10 hybrid structures with the lowest hybridization energy which are shown in Table 1 and Figure 1 which illustrate these miRNAs' binding locations around the matching UTR sequences were used to develop the secondary structure of the selected miRNAs and mRNAs. By sending potential UTR region sequences and matching miRNA sequences to the RNAfold web server, an ensemble of bimolecular structures was developed. Table 2 shows the dot‐bracket structure of these miRNA–mRNA complexes developed by RNAfold. The secondary structure of miRNA–mRNA interactions is depicted by dot‐bracket notation, which also displays base pairing and sequences. In the given table parentheses indicate paired bases, whereas dots indicate unpaired nucleotides. The structure of the miRNA–mRNA duplex is shown visually by each parenthesis, which represents a nucleotide pair. The base pairing patterns and overall secondary structure are indicated by the opposing pairs of parentheses. Predicted UTR sequence shape after miRNA hybridization shows a combination of hairpins, folds, and loops. While secondary structure prediction methods aim to identify the MFE structure of RNA sequences using the assumption that base pair stacking and loop entropies contribute additively to the energy of a nucleic acid secondary structure. Figures 2 and 3 showed the secondary structure of targeted miRNA–mRNA complex. Similar to proteins, miRNAs' function is determined by their structure, which is encoded in the sequential arrangement of nucleotides. Three‐dimensional (3D) models provide a more realistic portrayal and emphasize underlying biology, such as a miRNA's binding effectiveness to its mRNA targets [58].

**Table 2:** The table displays the projected miRNA–mRNA duplexes' dot‐bracket structures.

SI. No	MiRNA–mRNA	Dot bracket structure
1.	Protein A 3′ UTR: hsa‐let‐7a‐5p	(((((((….)))))))
2.	Protein A 5′ UTR: hsa‐mir‐193b‐5p	(((.(((((((((…..((((…)))))).))))))).)))
3.	ClfB‐3′ UTR: hsa‐mir‐29‐1‐5p	(((.((…(((((((((….))))))))).)))))
4.	ClfB‐5′ UTR: hsa‐mir‐31‐5p	………..((((((((……))))))))….
5.	HtrA5′ UTR: hsa‐mir‐6763‐5p	(((((((((……..)))))))))
6.	HtrA3′ UTR: hsa‐mir‐192‐3p	(((.(((((((((….)))))).))).)))
7.	MgrA3′ UTR: hsa‐mir‐128‐1‐5p	((((((((((………))))))))))
8.	MgrA5′ UTR: hsa‐mir‐30e‐3p	(((((((((….)))))))))
9.	SarA 5′ UTR: hsa‐mir‐34a‐5p	((((((((…..((((((….))))))…))))))))
10.	SarA 3′ UTR: hsa‐mir‐208b‐5p	….((((((((((……))))))))))…..

In comparison to 3D structure prediction approaches for proteins, only a few methods have been developed for RNA (including RNAComposer, ModeRNA, and MC‐SYM). The majority of these programs make 3D structure predictions using single‐stranded RNA sequences and secondary structure folding information in dot‐bracket notation. To mimic interacting RNA molecules (such as miRNA–mRNA duplexes), sequences are concatenated into a single strand and then separated by eliminating the phosphodiester linkages [59]. RNAComposer, tool was used to investigate the tertiary structure of the miRNA–mRNA duplex. This tool makes it possible to view and analyze intricate RNA structures in 3Ds. It provides the ability to forecast, display, and export tertiary structures for additional study. We analyzed the structure to determine important interactions, stability of the conformation, and functional consequences. The comprehension of the regulatory systems and possible treatment targets is aided by these insights. The stable bimolecular structures suggested by these advantageous conformations point to a higher potential for suppressing gene expression. The 3D structures of the mRNA–miRNA duplexes are shown as a part of pre‐ and post‐MD complexes in later part of this text.

### 3.3 MD Simulation of miRNA and mRNA Complex

**Figure 4:**
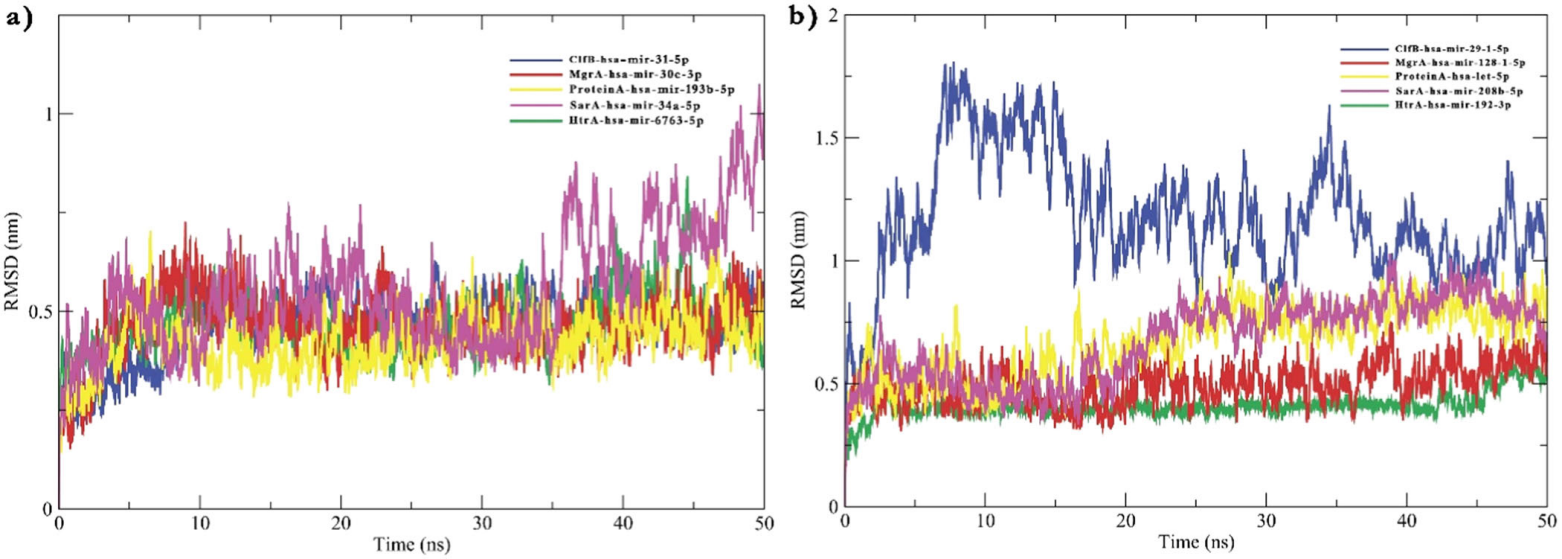
Root mean square deviation of miRNA–mRNA duplex: (a) The RMSD analysis of duplexes formed by miRNA annealing with 5′ UTR sequences of respective mRNA of virulent proteins. (b) RMSD analysis of mRNA–miRNA duplex formed due to miRNA binding to 3′ UTRs. ClfB is denoted in blue, MgrA is denoted in red, Protein A in yellow, SarA shown in magenta, and HtrA in green.

**Figure 5:**
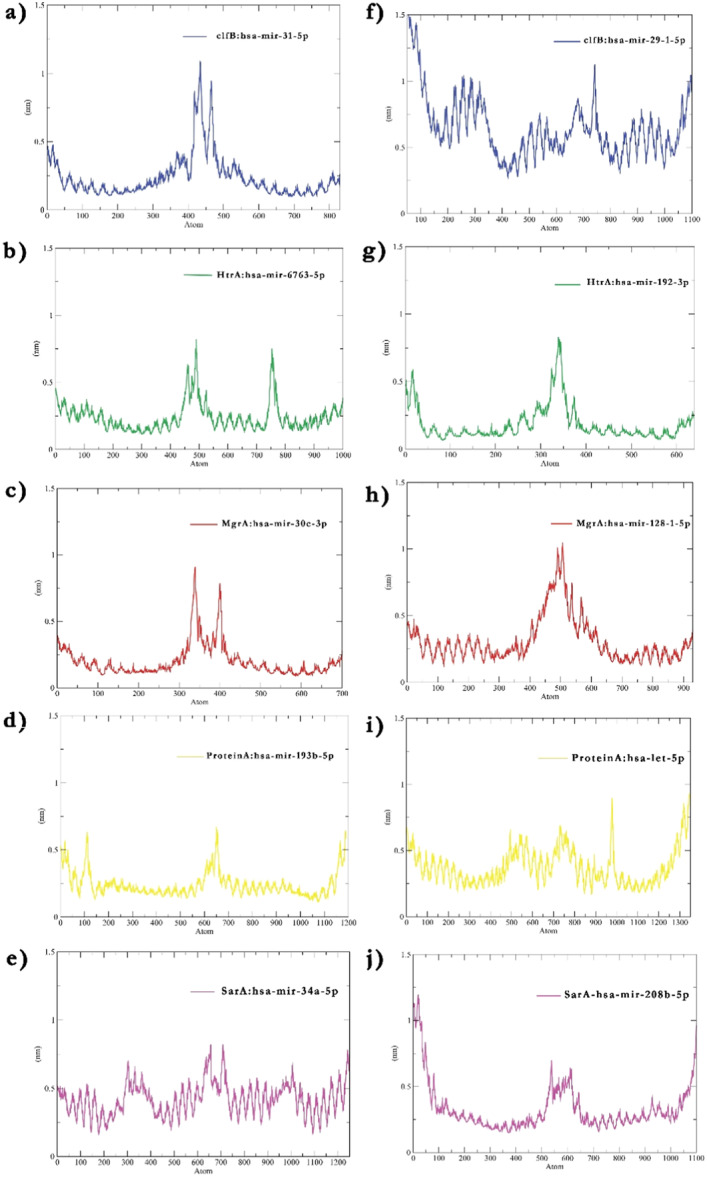
Root mean square fluctuation analysis at atomic level of mRNA–miRNA duplex: (a–e) RMSF calculated for duplexes created due to miRNA binding to 5′ UTRs. (f–j) RMSF calculated for duplexes created due to miRNA binding to 3′ UTRs. ClfB is denoted in blue, MgrA is denoted in red, Protein A in yellow, SarA is shown in magenta, and HtrA in green.

MD is a crucial tool to predict the fate of molecular interaction. In this work to GROMACS was deployed to analyze the stability of the mRNA–miRNA complex and to detect any unwinding events. The RMSD plots of complexes obtained on analyzing miRNA bound to 5′ UTR indicated the complexes achieved enough stability as suggested by minimal functions around 0.5 nm (Figure 4a). The SarA:hsa‐mir‐34a‐5P complex exhibited an increase in fluctuations after 35 ns which was certainly in contrast to other complexes formed with 5′ UTR of respective protein mRNA. On the other hand, complexes formed due to the binding of respective miRNAs with 3′ UTR of mRNA showed the complexes MgrA‐3′ UTR: hsa‐mir‐128‐1‐5p and HtrA‐3′ UTR: hsa‐mir‐192‐3p showed significantly stable interaction as suggested by lesser RMSD fluctuations limited to 0.5 nm (Figure 4b). A minimally higher fluctuation after 15 ns was observed in complexes Protein A‐3′ UTR: hsa‐let‐7a‐5p followed by SarA‐3'UTR: hsa‐mir‐208b‐5p where fluctuations reached 1.0 nm. ClfB‐3′ UTR: hsa‐mir‐29‐1‐5p in contrast to others obtained a significantly higher fluctuation in the RMSD plot from 0.9 to 1.7 nm. RMSF plot was generated to visualize the fluctuations of nucleic acid bases from their relative positions at an atomic level (Figure 5). The observations were found to perfectly complement the RMSD data. A thorough analysis of duplexes formed involving 5′ UTRs of corresponding mRNAs disclosed SarA‐5′ UTR: hsa‐mir‐34a‐5p duplex had a higher flexibility owing to its greater fluctuation in the RMSF plot (Figure 5e). This observation was unlike other mRNA–miRNA duplexes as they were found to be fluctuating around 0.25 nm stating a more stable complex formation. The sudden peaks observed in the middle of the RMSF plot were due to the fluctuating terminal nucleic acid bases, observed in all the duplexes and do not suggest any further instability. The duplexes formed due to miRNAs binding to 3′ UTR of the mRNAs displayed an all‐over higher RMSF with an average of 0.73 nm for ClfB‐3′ UTR: hsa‐mir‐29‐1‐5p (Figure 5f). All other duplex groups formed due to miRNA binding at 3′ UTRs did not disclose any significant fluctuations except the terminal bases. RMSF plot disclosed lesser plasticity in the HtrA‐3′ UTR: hsa‐mir‐192‐3p group as it showed far less variation in the relative atomic positions with an average of 0.18 nm (Figure 5g). The 0.33 nm average RMSF of SarA‐3′ UTR: hsa‐mir‐208b‐5p similarly indicated a stable interaction (Figure 5j).

**Figure 6:**
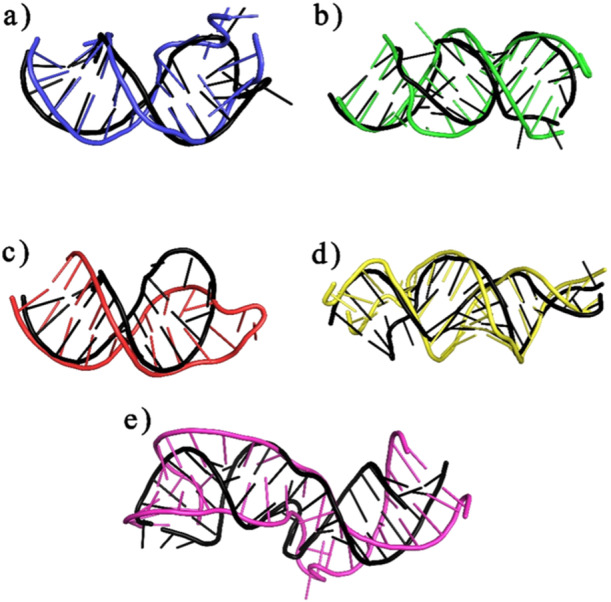
Pre‐ and post‐MD structures of mRNA–miRNA hybrids: The images denote the fate of MD simulation on nucleic acid duplexes formed after miRNA binding to 5′ UTRs of selected virulent protein mRNAs. None of the hybrids indicated any significant unwinding event. (a) ClfB‐5' UTR: hsa‐mir‐31‐5p duplex, (b) HtaA‐5' UTR: hsa‐mir‐6763‐5p duplex, (c) MgrA‐5' UTR: hsa‐mir‐30e‐3p duplex, (d) Protein A‐5' UTR: hsa‐mir‐193b‐5p duplex, and (e) SarA‐5' UTR: hsa‐mir‐34a‐5p. The pre‐MD duplexes for each group are shown in black. ClfB is denoted in blue, MgrA is denoted in red, Protein A in yellow, SarA is shown in magenta, and HtrA in green.

**Figure 7:**
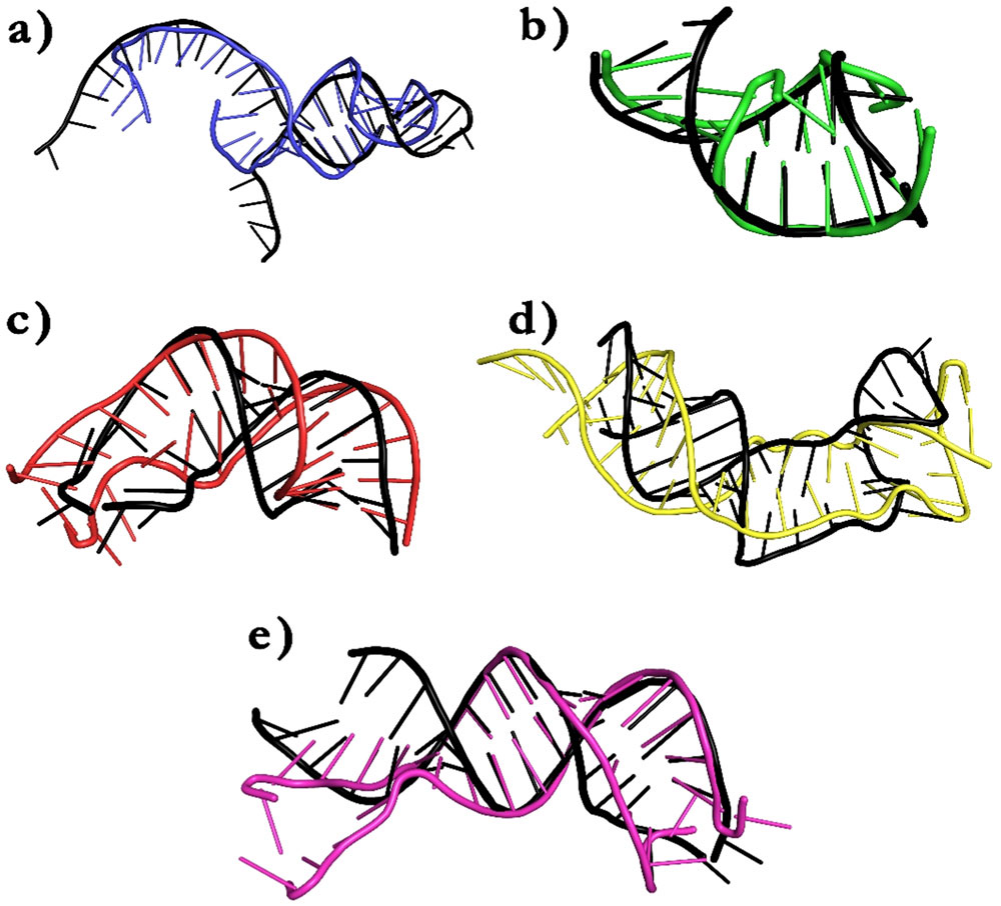
Pre‐ and Post‐MD structures of mRNA–miRNA hybrids: The images denote the fate of MD simulation on mRNA–miRNA duplexes built after miRNA binding to 3′ UTRs of selected virulent proteins. None of the hybrids indicated any significant unwinding event. (a) ClfB‐3' UTR: hsa‐mir‐29‐1‐5p, (b) HtrA‐3' UTR: hsa‐mir‐192‐3p, (c) MgrA‐3' UTR: hsa‐mir‐128‐1‐5p, (d) Protein A‐3' UTR: hsa‐let‐7a‐5p, and (e) SarA‐3' UTR: hsa‐mir‐208b‐5p. The pre‐MD duplexes for each group are shown in black. ClfB is denoted in blue, MgrA is denoted in red, Protein A in yellow, SarA is shown in magenta, and HtrA in green.

Furthermore to ascertain if any dissociation or unbinding event took place the pre‐ and post‐MD complexes were extracted. No significant unbinding was observed in any miRNA–mRNA duplexes throughout a 50 ns MD run (Figures 6 and 7).

## 4 Discussion

Understanding miRNA‐mediated gene regulation relies heavily on computational approaches such as miRNA structure prediction, target gene prediction, and gene regulatory network modeling. These computational techniques provide essential insights into miRNA activities, regulatory networks, and the evolutionary dynamics of bacterial systems. Our in‐silico hybridization work demonstrates the ability of human miRNAs to interact with and potentially influence the expression of virulence factor genes in *S. aureus*. By targeting both the 5′ UTR and 3′ UTR regions of these genes, we aimed to identify miRNAs that could reduce bacterial virulence, thereby opening new avenues for therapeutic interventions against gram‐positive bacterial infections.

Our research uncovered several miRNAs with significant hybridization potential to target mRNA sequences, demonstrating their ability to bind and decrease gene expression. Notably, hsa‐mir‐193b‐5p showed strong binding to the Protein A mRNA, with low hybridization energy values indicating a high binding affinity. These findings suggest that hsa‐mir‐193b‐5p, along with other identified miRNAs, may play a critical role in modulating *S. aureus* virulence by targeting specific virulence components. The reliability of our results was ensured by utilizing the use of dual‐energy thresholds, namely IntaRNA (−9 kcal/mol) and RNAhybrid (−22 kcal/mol). We were able to discover miRNAs with low hybridization energies and high binding affinities due to these thresholds. For instance, hsa‐mir‐193b‐5p's minimal free energy (mfe) of −29 kcal/mol with a combined free energy *E* of −18.46 kcal/mol, a hybridization energy of −21.49 kcal/mol for IntaRNA, indicating that it may be effective in targeting the ProteinA mRNA. The MFE represents the stability of miRNA–mRNA interactions in addition to indicating to the secondary structure that is most energetically favorable. The total access energy (*E*), which includes mfes and hybridization energies, plays a critical role in both accurately predicting miRNA targets and comprehending the regulatory interactions between miRNAs and mRNAs. Stronger miRNA–mRNA interactions are indicated by more stable and energetically advantageous structures, which are indicated by lower mfe and hybridization energy values. For gene regulation to work effectively, this stability is essential. On the other hand, hsa‐mir‐29‐1‐5p and hsa‐mir‐31‐5p, two miRNAs that target ClfB mRNA, showed the least effectiveness. Their respective RNAhybrid mfes were −23.7 and −22.3 kcal/mol, hybridization energies of −12.01 and −15.52 kcal/mol, with total energies of −9.2 and −9.99 kcal/mol, respectively, were obtained via IntaRNA analysis. These findings imply that these miRNAs have less success in binding to the ClfB mRNA. Though simulating organic molecules in liquid phase only provides theoretical data but it also discloses certain insights not revealed by experimental data. MD has the potential to identify functionally relevant conformations, which sometimes can be concealed during experimental techniques. Besides it also provides the details of transitions between these conformations. MD simulation considers the effect of solvent model and the temperature unraveling its effect on simulated macromolecules giving us an idea on probable fate [60, 61].

The RNAfold online tool helped us predict the secondary structures of miRNA–mRNA duplexes, which are crucial for understanding the stability and functionality of these interactions. Our research identified stable secondary structures, indicating robust miRNA–mRNA interactions. Furthermore, the RNAComposer tool provided insights into the tertiary structures, allowing us to examine and evaluate the complex 3D conformations of these duplexes. These structural insights are essential for understanding the functional implications of miRNA binding and the potential inhibition of gene expression.

MD simulations showed that most of the predicted miRNA and mRNA duplexes could form stable interactions. The MD results were complementary to the binding energy data obtained from IntaRNA and RNAhybrid servers. For instance, Protein A‐5′ UTR hybrids, which had the lowest mfe, showed modest RMSD fluctuation in the MD trajectory. The low‐level RMSD fluctuation suggested negligible structural changes and lesser plasticity. Similarly, hsa‐mir‐30e‐3p and hsa‐mir‐128‐1‐5p binding to the 5′ and 3′ UTR of MgrA mRNA displayed overall decent interaction, with the 3′ UTR duplex achieving a higher hybridization score. On the other hand, ClfB‐3′ UTR showed inferior binding energy in both RNAhybrid and IntaRNA implying a weaker interaction. The RMSD plot for this duplex showed significantly higher fluctuation than other hybrids, suggesting structural changes. The RMSF plot also indicated more structural plasticity for the ClfB‐3′ UTR duplex due to greater deviation of nucleic acid bases from the mean position. The pre‐MD and post‐MD snapshots revealed many unpaired bases in the said duplex, which may contributed to weaker complex formation. A low‐affinity interaction was also noted for Protein A‐3′ UTR, where both RNAhybrid and IntaRNA scores were supported by higher RMSD and RMSF values from MD. The RMSD kept increasing after 15 ns, and a higher average RMSF of 0.37 nm was observed. Theoretically, the hybrids ClfB‐3’ UTR and SarA‐5′ UTR may have lower chances of forming stable duplexes compared to others, while Protein A‐5'UTR, HtrA‐3′ UTR, and HtrA‐5′ have a greater propensity to form strong interactions. Some discrepancies between MD data and binding energy output were observed for SarA‐5′ UTR, where some instability was noted in the RMSD curve after 40 ns, although RMSF could not uncover any serious atomic fluctuations.

The in‐silico approach has provided valuable insights into potential human miRNAs that could inhibit the expression of virulent proteins in *S. aureus*, offering an efficient platform for further experimental validation. The use of miRNAs to target bacterial virulence factors is a promising research area. Notably, miRNAs function similarly to small RNAs (sRNAs), by sharing several mechanistic similarities despite their differences in evolution. Both miRNAs and sRNAs regulate gene expression by binding to specific target mRNAs, leading to their degradation or inhibition of translation [62, 63]. Therefore, research on sRNA‐mediated gene regulation in *S. aureus* can provide a useful foundation for exploring miRNA‐based therapeutic strategies.

The delivery of genetic materials to gram‐positive bacteria can be complicated but newer methods have been developed to streamline the process. Cell Penetrating Peptides (CPPs) are proven to be highly effective for transporting various biomolecules, including miRNAs, into cells [64]. CPPs are positively charged due to their high arginine and lysine content, and their hydrophilic nature allows them to cross cell membranes efficiently [65]. Since bacterial surfaces tend to carry a net negative charge, primarily due to acidic polysaccharides, teichoic acids, and lipoteichoic acids, conjugating miRNAs with CPPs could provide an effective strategy for their delivery into bacterial cells [66, 67]. In addition, electroporation is a widely used method for transformation of cells including bacteria by creating transient pores in the cell membrane [68].

Successful delivery of miRNAs to bacterial cells could result in the downregulation of virulent factor production, offering a potential therapeutic strategy to reduce *S. aureus* virulence and improve clinical outcomes for patients suffering from the disease. This approach may serve as an alternative to conventional antibiotics, particularly in light of the growing threat of antibiotic resistance.

However, the dynamic nature of bacterial genomes and their rapid mutation rates pose additional challenges. Future research must account for the genetic diversity among different *S. aureus* strains to ensure that the identified miRNAs can target a broad range of bacterial variants.

## 5 Conclusion

The current study underscores the potential of in‐silico hybridization and MD simulations as powerful tools for identifying novel miRNA candidates that target virulent proteins in *S. aureus*, which suggests a promising avenue for developing miRNA‐based therapies. By identifying miRNAs with high binding affinity and analyzing their secondary and tertiary structures, we have laid the groundwork for therapeutic applications. The use of MD simulations was instrumental, providing deep insights into the stability and interaction dynamics of miRNA‐target complexes at an atomic level. These simulations have made it possible to predict binding affinities and conformational changes, enhancing the comprehension of miRNA‐target interactions.

The integration of MD simulations not only strengthens the validity of our findings but also introduces a novel approach to developing miRNA‐based therapies. The results present a potential strategy for combating bacterial infections and addressing the escalating issue of antibiotic resistance. This could ultimately lead to innovative treatments for bacterial infections that are increasingly resistant to conventional antibiotics.

## Author Contributions

H.T. contributed to conceptualization, performing computational work, and preparing the manuscript. S.S. contributed to molecular dynamics simulation and manuscript preparation. M.G. supervised the work and contributed to reviewing and editing the draft.

## Conflicts of Interest

The authors declare no conflicts of interest.

## Data Availability

The data that support the findings of this study are available from the corresponding author upon reasonable request.
